# Synergistic effects of Smac mimetic APG-1387 with anti-PD-1 antibody are attributed to increased CD3 + NK1.1 + cell recruitment secondary to induction of cytokines from tumor cells

**DOI:** 10.1186/s12935-024-03373-7

**Published:** 2024-05-24

**Authors:** Wentao Pan, Qiuyun Luo, Eric Liang, Mude Shi, Jian Sun, Huimin Shen, Zhenhai Lu, Lin Zhang, Xianglei Yan, Luping Yuan, Suna Zhou, Hanjie Yi, Yifan Zhai, Miao-zhen Qiu, Dajun Yang

**Affiliations:** 1 Department of Experimental Research, State Key Laboratory of Oncology in South China, Collaborative Innovation Center for Cancer Medicine, Sun Yat-sen University Cancer Center, Guangzhou, China; 2 Ascentage Pharma (Suzhou) Co, Ltd, Suzhou, Jiangsu Province China; 3 Department of Clinical Research Center, The Third Affiliated Hospital of Sun Yat-sen University, Guangzhou, Guangdong China; 4 Department of Gynecology, The First Affiliated Hospital of Sun Yat-sen University, Guangzhou, China; 5 Department of Colorectal Surgery, Sun Yat-Sen University Cancer Center, Guangzhou, China; 6 Department of Clinical Laboratory, Sun Yat-sen University Cancer Center, Guangzhou, China; 7 Department of Medical Oncology, State Key Laboratory of Oncology in South China, Collaborative Innovation Center for Cancer Medicine, Sun Yat-Sen University Cancer Center, Guangzhou, China

**Keywords:** Smac mimetic, APG-1387, Combination therapy, Immunotherapy, CD3 + NK1.1 + cells

## Abstract

**Background:**

Immune checkpoint inhibitors are approved for the treatment of various tumors, but the response rate is not satisfactory in certain malignancies. Inhibitor of apoptosis proteins (IAP) ubiquitin-E3 ligase activity is involved in the regulation of immune responses. APG-1387 is a novel second mitochondria-derived activator of caspase (Smac) mimetic IAP inhibitor. The aim of this study was to explore the synergistic effect of APG-1387 when combined with anti-PD-1 antibody in a preclinical setting.

**Methods:**

We utilized syngeneic mouse models of ovarian cancer (ID8), colon cancer (MC38), malignant melanoma (B16), and liver cancer (Hepa1-6) to assess the combination effect of APG-1387 and anti-PD-1 antibody, including immune-related factors, tumor growth, and survival. MSD V-PLEX validated assays were used to measure in vitro and in vivo cytokine release.

**Results:**

In ID8 ovarian cancer and MC38 colon cancer models, APG-1387 and anti-PD1 antibody had synergistic antitumor effects. In the MC38 model, the combination of APG-1387 and anti-PD-1 antibody significantly inhibited tumor growth (*P* < 0.0001) and increased the survival rate of tumor-bearing animals (*P* < 0.001). Moreover, we found that APG-1387 upregulated tumor-infiltrating CD3 + NK1.1 + cells by nearly 2-fold, by promoting tumor cell secretion of IL-12. Blocking IL-12 secretion abrogated the synergistic effects of APG-1387 and anti-PD-1 antibody in both MC38 and ID8 models.

**Conclusions:**

APG-1387 has the potential to turn “cold tumors” into hot ones by recruiting more CD3 + NK1.1 + cells into certain tumors. Based on these and other data, the safety and therapeutic effect of this combination will be investigated in a phase 1/2 trial in patients with advanced solid tumors or hematologic malignancies (NCT03386526).

**Graphic abstract:**

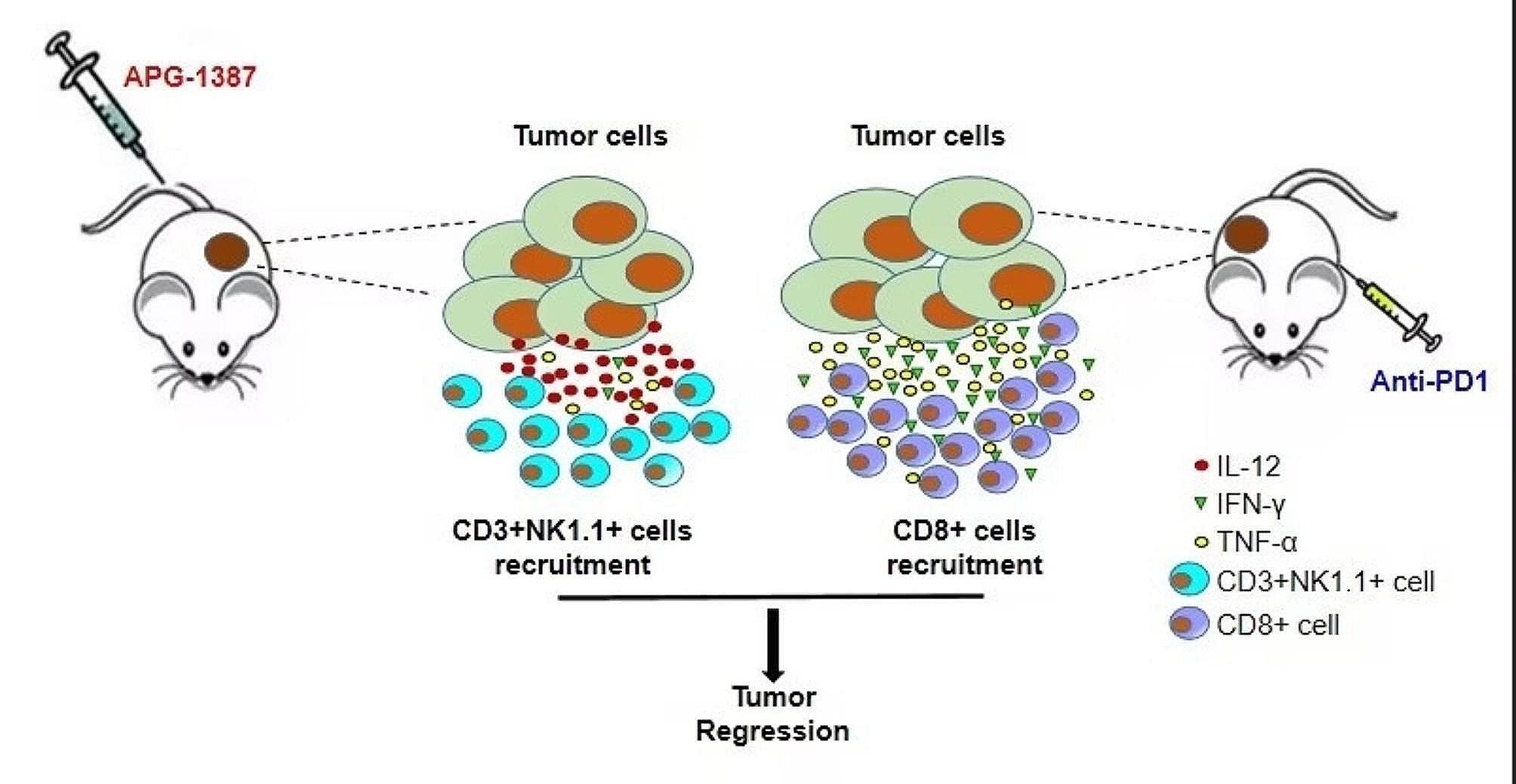

**Supplementary Information:**

The online version contains supplementary material available at 10.1186/s12935-024-03373-7.

## Introduction

Direct stimulation of the immune system with immune checkpoint inhibitors (ICIs), such as antibodies against programmed death protein 1 (PD-1) and its ligand, programmed death ligand 1 (PD-L1), has been reported in the management of multiple cancers [1–3]. Although these immunotherapies have transformed the treatment landscape for a range of solid tumors and other malignancies, certain cancers do not respond, such as microsatellite stable (MSS) colorectal carcinoma (CRC) and ovarian cancer [4]. There is hence an unmet medical need to identify strategies that may increase treatment responses. In the past 5 years, thousands of clinical strategies have failed to demonstrate synergistic effects of combination regimens including anti-PD-1 antibodies, despite frequently strong mechanistic rationales [5].

Inhibitors of apoptosis proteins (IAPs) are a class of proteins that negatively regulate caspases and apoptosis [6]. IAPs are frequently dysregulated in many cancers and are believed to underlie their resistance to chemotherapeutics [7–9]. In addition, these proteins are reported to play an important role in immune regulation [10], including ubiquitin (Ub)-dependent pathways that modulate innate immune signaling via activation of nuclear factor-κB (NF-κB) [11].

Based on their significant biological functions in cell death and immune responses, several IAP inhibitors have been developed, and their clinical synergistic effects with anti-PD1 therapies have been tested, although results were seldom reported [12–15]. Among the studies, a prolonged partial response (PR) in patients with MSS CRC has been observed at the lowest dose cohort during dose escalation of IAP inhibitor birinapant combined with a fixed dose of pembrolizumab. However, high-dose birinapant failed to meet its efficacy endpoints at the recommended phase 2 dose (R2PD), resulting in the termination of the phase 2 trial [12].

APG-1387 is a potent bivalent second mitochondria-derived activator of caspases (SMAC mimetic), a small molecule IAP inhibitor that was developed by Ascentage Pharma as a therapy for patients with cancer [16]. A phase 1 clinical trial in patients with solid tumors in China and the US shows minimal efficacy but a good safety profile ranging from 0.3 to 60 mg [17, 18].

Antiproliferative and antitumor activities of APG-1387 have been well documented in studies of a panel of cancer cell lines and xenograft tumor models [19–21]. Our previous study demonstrated that APG-1387 exhibited dual antitumor effects on PLC/PRF/5 hepatoma cells, not only directly inducing tumor cell apoptosis through the caspase pathway but also enhancing innate antitumor immunity by upregulating CD45 + NK1.1 cells [16]. APG-1387 also positively regulated T cells by reducing Treg-cell differentiation and downregulating PD-1 expression on CD4^+^ T cells.

Based on these findings suggesting that APG-1387 regulates multiple aspects of antitumor immunity, it is a promising candidate to potentiate the effects of a ICIs such as an anti-PD-1 antibody when the two agents are combined. Therefore, we explored the potential synergistic antitumor effect of APG-1387 combined with an anti-PD-1 antibody in several preclinical animal tumor settings. The aims of this study were to determine whether: (1) the combination of APG-1387 and anti-PD-1 antibody resulted in synergistic antitumor effects; and (2) if present, and the effects were dose-independent and model-dependent, what the main mechanisms of action.

## Materials and methods

### Chemicals

APG-1387 was provided by Ascentage Pharma Group Inc (Taizhou, China). For *in-vitro* assays, the compound was dissolved in dimethyl sulfoxide (DMSO; Sigma Aldrich, St. Louis, MO, USA) at a stock concentration of 40 mM and stored at -20℃. The final concentration of DMSO to dilute compound in culture media did not exceed 0.1%. For an *in-vivo* study, APG-1387 was formulated in 5% Cremophor EL (GBCBIO, Guangzhou, China), 10% polyethyleneglycol-400 (PEG-400; GBCBIO), 85% normal saline (GBCBIO). Anti-PD-1 antibody (BE0273, Bio X Cell, Lebanon, NH, USA) and IgG antibody (BE0089, Bio X Cell) were purchased from Univ-Bio Group Inc (Shanghai, China).

### Cell cultures

Mouse syngeneic tumor cell lines were purchased from American Type Culture Collection (ATCC, Manassas, VA USA) provided by Cobioer, including murine colon cancer cell line MC38 and CT26, murine liver cancer cell line Hepa1-6, and murine melanoma cell line B16. Murine ovarian cancer cell line ID8 with luciferase tag was a gift from Professor Xie Dan’s laboratory. Peripheral blood mononuclear cells (PBMC), MC38 cells and mouse splenocytes, were cultured in 1640 medium supplemented with 10% fetal bovine serum (FBS). Hepa1-6, ID8-luciferase, and B16 cells were cultured in Dulbecco’s modified Eagle’s medium (DMEM) containing 10% FBS. All culture media contained 100 IU/mL penicillin and 100 mg/mL streptomycin, and all cell culture reagents were purchased from Gibco™ (Thermo Fisher Scientific, Waltham, MA). All cells were maintained at 37 °C in 5% CO_2_.and all experiments were performed in the logarithmic growth phase of the cells.

Immune cells were extracted from mouse spleens, draining lymph nodes, and tumors. After mouse spleens and draining lymph nodes were ground in 1% FBS in Phosphate buffer saline (PBS), cells were centrifuged at 1,400 rpm for 5 min (min). The red blood cell lysate was added for 10 min, then cells were washed twice with PBS and filtered by a 70-µm cell sieve for staining.

Subcutaneous mouse tumors were placed into 5 ml PBS containing 2% FBS and cut into small pieces, and then washed with PBS. Tumors were resuspended in 15 mL RPMI supplemented with 2% FBS, 50 U/mL collagenase type IV (Invitrogen; Thermo Fisher, Waltham, MA), and 20 U/mL DNase (Roche Diagnostics; Swiss); samples were incubated at 37 °C for 2 h. Suspensions were washed three times with PBS and passed through a 70-µm strainer. PBMCs were isolated by Ficoll gradient centrifugation and collected for staining. For the ID8 model, artificial ascites was established for mechanism studies. First, 1 mL of PBS was injected into the ID8-bearing mouse’s abdomen, most was aspirated after irritation, the supernatant was collected for a cytokine study, and the cell precipitation collected for flow analysis. Another 3 mL PBS was then injected into the abdomen and all aspirated for cell collection. Finally, the cells were collected from the abdominal irritation and analyzed by flow cytometry.

### Antibodies used in flow cytometry analysis

Detailed information on the antibodies used in flow cytometry analysis is listed in supplementary material Table S1. All of antibodies were used at a 1:200 dilution.

### Intracellular cytokine staining

For intracellular detection of cytokines on lymphocytes, cells were first stained for extracellular markers, then suspended the cells in Fixation/Permeabilization reagent (eBioscience) for 18 h at 4 °C. Cells were then stained with an antibody against IFN-γ, washed in Permeabilization/Wash buffer (eBiosciences), and analyzed.

### Sample acquisition

Data were acquired on a Cytoflex (Beckman Coulter, Fullerton, CA, USA) and analyzed with FlowJo™ v107 (BD Biosciences, Ashland, OR, USA).

### Cell apoptosis detection

Apoptotic cells were identified using an Annexin V-FITC apoptosis detection kit (Neobioscience, China) and analyzed by flow cytometry. MC38 cells were treated with the indicated concentration of APG-1387 for 48 h, followed by harvesting, washing with cold PBS, and resuspension in 200 µL of 1× binding buffer. Annexin V-FITC and propidium iodide (PI) staining buffer were added and incubated at room temperature in the dark for 15 min. The samples were then diluted to a final volume of 400 µL/assay with ice-cold 1× binding buffer before analysis by FACS.

### Human PBMC collection

For PBMC extraction from whole blood cell, lymphocytes were isolated by density-gradient centrifugation. In brief, the blood sample was (1) diluted once with phosphate-buffered saline (PBS; sterilized, cell culture grade, Gibco) in a 1:1 ratio; (2) laid on the top of the same volume of Ficoll (p9011, Solarbio, Beijing, China) in a Falcon tube; and (3) centrifuged at room temperature at 400 g (relative centrifugal field [RCF], without brake) for 30 min. The interface (lymphocytes) was carefully removed to a new tube, washed with 10 mL of 1 × PBS, centrifuged at 500 g for 10 min; and resuspended with 1 to 5 mL of 1 × PBS to determine the viability and cell number of PBMCs.

### In vivo treatment of mouse syngeneic tumor models

All experiments were performed with Animal Ethics approval and conformed to regulatory standards set forth by the Sun Yat-sen University (SYSU) Animal Ethics Committee. All mouse strains were maintained in the specific-pathogen-free Laboratory Animal Center of SYSU. Female C57/B6 mice were purchased from Beijing Vital River Laboratory (Beijing, China) and treated at between 7 and 12 weeks of age. MC38 cells (5 × 10^5^), B16 cells (2 × 10^5^), and Hepa1-6 cells (1 × 10^6^) were resuspended in 100 µL of cold PBS and then subcutaneously injected into the dorsal flank of C57/B6 mice to establish colon cancer, melanoma, and liver cancer models, respectively. For the syngeneic ovarian cancer model, ID8-luciferase cells (6 × 10^6^) were intraperitoneally (IP) injected into mice. For drug efficacy studies in subcutaneous cancer models, mice were randomized into four groups of seven mice each with approximately equivalent tumor volumes when mean values reached approximately 50 to 100 mm^3^. APG-1387 0.2 mg/kg or vehicle was administered intravenously (IV) once every 3 days, while 100 µg/mouse anti-PD-1 antibody (BE0273, Bio X Cell, Lebanon, NH, USA) or vehicle and IgG antibody (BE0089, Bio X Cell) was administered by IP injection, also once every 3 days, two times in total. For interleukin-12 (IL-12) *in-vivo* blockade, 500 µg/mouse anti-IL12p40 antibody (BE0051, Bio X Cell) was introduced by IP injection two times (once every 3 days).

For MC38, B16 and Hepa1-6 models, the diameter and width of the tumors were measured twice per week and tumor volumes were calculated as V (mm^3^) = 1/2 × (length × width^2^). Mice were euthanized when tumor volumes reached 2,000 mm^3^. For the ID8 tumor model, bioluminescence was adopted to estimate tumor growth using an IVIS® Spectrum in vivo imaging system (PerkinElmer, Waltham, MA USA) and, on the tenth day after inoculation, mice with approximately the same photon flux were selected to treat. Tumor growth was monitored via IVIS every week until the presence of ascites. Mice were euthanized at the presence of ascites and severe hair loss.

### RNA sequencing

RNA sequencing was performed by Annoroad Gene Technology (Beijing, China) after PBMC RNA extraction using an RNAprep pure Cell/Bacteria Kit (TIANGEN, Beijing, China). A genetic library was established under standard protocol and sequenced using the Illumina (San Diego, CA, USA) platform according to a PE150 sequencing strategy. The sequence of each sample was compared with the reference genome, using. HISAT2 (hierarchical indexing for spliced alignment of transcripts) is the engine to conduct alignment analysis. (HISAT2 is a rapid and sensitive alignment program to map next-generation sequencing “reads,” including either RNA or DNA, to a single reference, or population of, human genomes.)

FPKM (fragments per kilobase of exon per million mapped fragments) represents a very effective tool for quantitatively estimating gene expression values using RNA-Seq technology. Gene differential expression analysis was performed using DEGseq, the treatment and reference groups were compared, the genes of |log_2_ratio| ≥ 1 and q < 0.05 were selected as significant differentially expressed genes, and the number of upregulated genes was obtained.

In the absence of biological replicates, DEGseq [22] was used for gene differential expression analysis. The treatment and reference groups were compared, and the genes of |log_2_ratio|≥1 and q < 0.05 were selected as significantly differentially expressed genes in order to obtain the number of genes up and down. DEGseq is based on the assumption that the count value is binomially distributed. An application of Bland-Altman plots to visually represent genomic information, MA plots visualize differences between measurements taken in two samples by transforming them onto log ratio (M) and mean average (A (mean average) scales, and then plotting these values. Based on MA plot theory, the approximate normal distribution of the M values ​​is estimated, and the probability (*P*) value of each gene in the two samples without expression difference is calculated, with correction by the Benjamini or Storey method to obtain the q value (false discovery rate). NCBI (National Center for Biotechnology Information), Uniprot, GO (Gene Ontology), and KEGG (Kyoto Encyclopedia of Genes and Genomes) databases were used to annotate differentially expressed genes and obtain their detailed description information.

### Meso scale discovery (MSD) multicytokine assay

Ten cytokines were detected using the V-PLEX kit (K15048D, MSD) and MSD QuickPlex SQ120 (Meso Scale Diagnostics, Rockville, MD, USA) according to kit instructions. Because MSD is based on electrochemiluminescence technology, the signal molecule SULFO-TAG can generate signals only under the condition of electric excitation. Therefore, the entire experimental apparatus does not need to be protected from light and is not affected by differences in the technical levels of the experimental operators.

### Analysis of the expression correlation between IAP2 (cIAP2) and PD-L1

The Gene Expression Profiling Interactive Analysis (GEPIA 2.0, http://gepia2.cancer-pku.cn/#index) web server represents a high-quality resource for gene expression analysis utilizing tumor and normal samples sourced from The Cancer Genome Atlas (TCGA) and Genotype-Tissue Expression (GTEx) databases. The correlation between IAP2 (BIRC3) and PD-L1 (CD274) gene expression in ovarian cancer was carried out using TCGA data through GEPIA 2.0 online analysis.

### Statistical analysis

Statistical analyses were performed using GraphPad Prism software v6.0 (GraphPad, La Jolla, CA). Unless otherwise indicated, results indicate mean ± SD of three independent experiments. Differences between the two groups were analyzed using unpaired sample t-tests. Differences between more than two groups were analyzed using a one-way analysis of variance (ANOVA) and two-way ANOVA. The Kaplan-Meier method was used to plot survival curves, and a log-rank test was used to compare survival differences between groups. A two-tailed α = 0.05 was considered statistically significant, with the following annotations of significance: *P < 0.05, **P < 0.01, and ***P < 0.001.

## Results

### The combination of APG-1387 and anti-PD-1 antibody inhibits tumor growth in ID8 ovarian cancer model

**Fig. 1:**
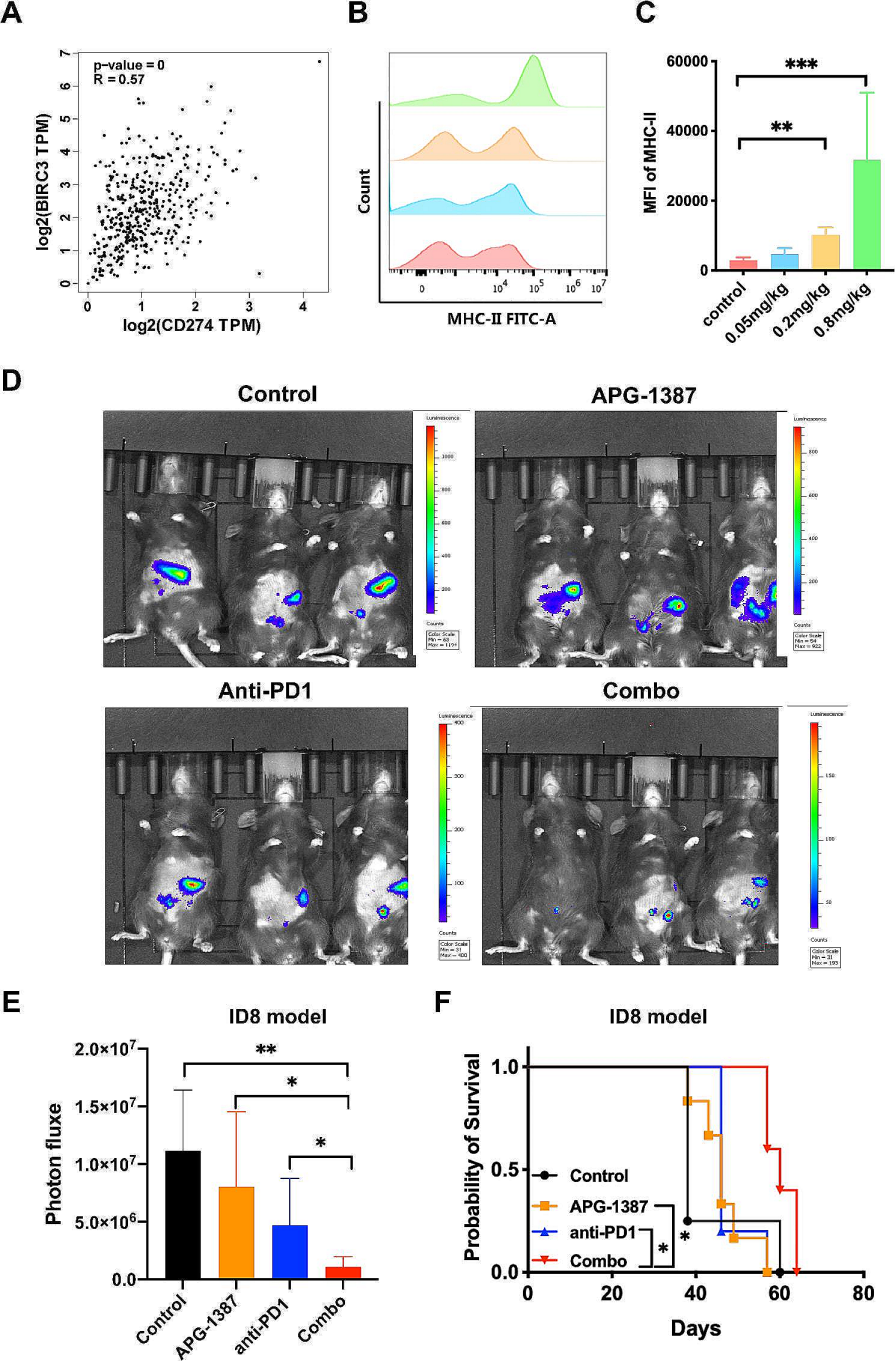
APG-1387 combined with anti-PD-1 antibody significantly inhibits growth of ID8 ovarian cancer in vivo. **(A)** Correlation of cIAP2(baculoviral IAP repeat containing 3 [BIRC3]) and PD-L1(CD274) in ovarian cancer was generated in Gene Expression Profiling Interactive Analysis website (GEPIA 2.0, http://gepia2.cancer-pku.cn/#index). **(B)** Expression of MHC-II in splenocytes detected by fluorescence-activated cell sorting (FACS) after treatment with indicated doses of APG-1387 or vehicle for 7 days. **(C)** Quantitation of median fluorescent intensity (MFI) of MHC-II in Fig. 1B. **(D)** Representative bioluminescence imaging of ovarian cancer cells ID8-bearing mice treated with IgG and vehicle control, APG-1387, anti-PD-1 antibody, or the APG-1387 and anti-PD-1 antibody combination (“Combo”) in 7 days after the final treatment. **(E)** Comparison of bioluminescence photon flux measured by an in vivo imaging system (IVIS) in four groups 7 days after the final treatment (6 mice per group). **(F)** Comparison of survival curves across the four treatment groups within the ID8 ovarian cancer model (6 mice per group). All data are shown as the mean ± SD. Statistical analyses were performed using one-way ANOVA, *P < 0.05, **P < 0.01, ***P < 0.001

Our previous studies [19] demonstrated that APG-1387 promoted apoptosis and autophagy in ovarian cancer cells. Using the GEPIA 2.0 online analysis website, we found a positive correlation between IAP2 (cIAP2) and PD-L1 expression in ovarian cancer within the TCGA database (Fig. 1A). Therefore, we speculated that targeting cIAP2 might downregulate PD-L1 and overcome tumors to evade immune surveillance. To determine the *in-vivo* dose of APG-1387, we explored doses of 0.05, 0.2, and 0.8 mg/kg of APG-1387 in a wild-type C57 model and found that expression of MHC-II in splenocytes was enhanced in a dose-dependent manner, with a significant difference emerging at the 0.2-mg/kg dose (Fig. 1B-C). Therefore, 0.2 mg/kg of APG-1387 was selected for further experiments.

Next, we intraperitoneally injected ID8-luciferase cells into C57/B6 mice with intact immune systems to establish an ovarian cancer model and then to evaluate the anti-tumor effects of APG-1387 in combination with anti-PD1 antibody. And then the mice were randomly divided into four groups to receive vehicle and IgG control, APG-1387 monotherapy, anti-PD-1 antibody monotherapy, and a combination of two drugs. As shown in Fig. 1D-E, the growth of the tumor was monitored using bright field and IVIS Lumina imaging, revealing that the combination therapy significantly delayed the tumor growth compared to individual treatment alone. The combination treatment significantly prolonged survival compared to the single agent group (Fig. 1F).

### APG-1387-induced release of IFN-γ, IL-5, IL-12p70, and TNF-α from tumor cells is necessary for the synergistic effect between APG-1387 and anti-PD-1 antibody

**Fig. 2:**
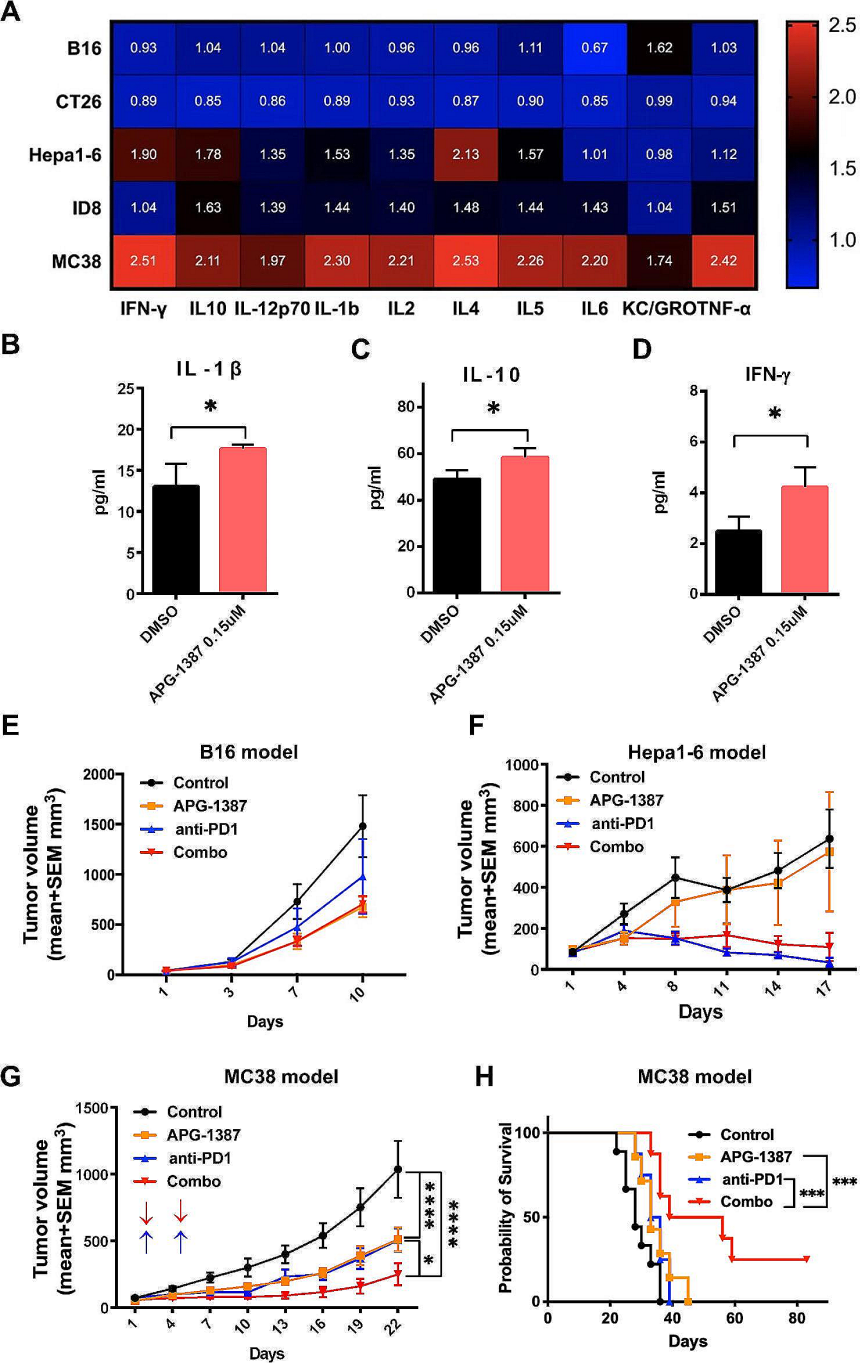
Cytokine release induced by APG-1387. **A** Heat-map representation of 10 mouse cytokine levels in cell supernatant collected from five mouse tumor cell lines treated with APG-1387 at 0.15 µM after 48 h, shown normalized to mean values of three vehicle-treated controls. Color scale from 0 to 2.5 induction is shown. **B-D** Cytokine (MSD method) analysis of IL-1β, IL-10 and IFN-γin C57 splenocytes treated with DMSO or APG-1387 for 48 h in triplicate. All data are shown as the mean ± SD. Statistical analyses were performed using unpaired t-test. *P < 0.05. **E** Tumor growth of B16 melanoma model treated with IgG and vehicle control, APG-1387, anti-PD-1 antibody and the combination of these. **F** Tumor growth of Hepa1-6 model treated with IgG and vehicle control, APG-1387, anti-PD-1 antibody, and the combination. **G** Tumor volumes of MC38 across the four groups (*n* = 7) were measured at indicated days. Data are shown as mean ± SEM of each group. *P < 0.05 when the combination group was compared to the anti-PD1 antibody group; ****P < 0.0001 when the APG-1387 group, anti-PD1 antibody group, and combination group were compared to the IgG and vehicle control group (calculated by two-way ANOVA with Tukey’s multiple comparisons test at day 22 after treatment onset). **H.** Comparison of survival curves across the four groups in Fig. 2G. ***P < 0.001 by log-rank test. Median survival for IgG and vehicle control, APG-1387, anti-PD-1 antibody, and combination groups were 28, 33, 34.5, and 47.5 days, respectively

To explore the mechanism of the synergistic effect between APG-1387 and anti-PD-1 antibody, we treated ID8 cells with 0.15 µM APG-1387 for 48 h and detected the levels of 10 common cytokines (IL-12p70, IFN-γ, IL-2, IL4, IL5, IL6, KC/GRO and TNF-α) by the MSD method in cell culture supernatant. APG-1387 induced ID8 to release more IFN-γ, IL-5, IL-12p70, and TNF-α (Fig. 2A), which were necessary for T-cell proliferation and function.

Next, we wonder whether these cytokines instrumental to the synergistic effect between APG-1387 and the anti-PD-1 antibody. To answer this question, we assessed another four cancer cells within the same experimental setting, including MC38 and CT26 colon cancer, B16 melanoma, and Hepa1-6 hepatoma. A similar pattern of changes in cytokines was observed in MC38 and Hepa1-6, but not the other two cell lines (Fig. 2A). Meanwhile, APG-1387 stimulated quiescent C57 splenocytes to express more IL-1β, IL-10, and IFN- γ (Fig. 2B-D).

In order to further investigate the in vivo antitumor efficacy of APG-1387 and anti-PD-1 antibody across different tumor types, B16 melanoma, Hepa1-6 hepatoma carcinoma, and MC38 colon cancer models were established in C57/B6 mice. The mice-bearing tumors were randomly divided into four groups and administered with either the vehicle control, APG-1387, anti-PD-1 antibody, or a combination of both. Throughout the duration of the experiment, tumor volume was measured in each group to assess antitumor activity. In the B16 melanoma model and Hepa1-6 hepatoma carcinoma model, the combination of APG-1387 and anti-PD-1 antibody did not demonstrate a synergistic antitumor effect compared to the single agent group (Fig. 2E-F). However, in the MC38 colon cancer model, APG-1387 combined with anti-PD-1 antibody generated a much higher anticancer activity compared with that of either drug alone, and prolonged mouse survival (Fig. 2G-H). One-quarter of the mice in the combination group were cured. These results indicate that APG-1387-stimulated release of IFN-γ, IL-5, IL-12p70, and TNF-α from the tumor were necessary but not sufficient for the synergistic effect between APG-1387 and anti-PD-1 antibody.

### APG-1387-induced CD3 + NK1.1 + cells infiltration in tumor tissue plays an important role in mediating the synergistic effect of APG-1387 and anti-PD-1 treatments

**Fig. 3:**
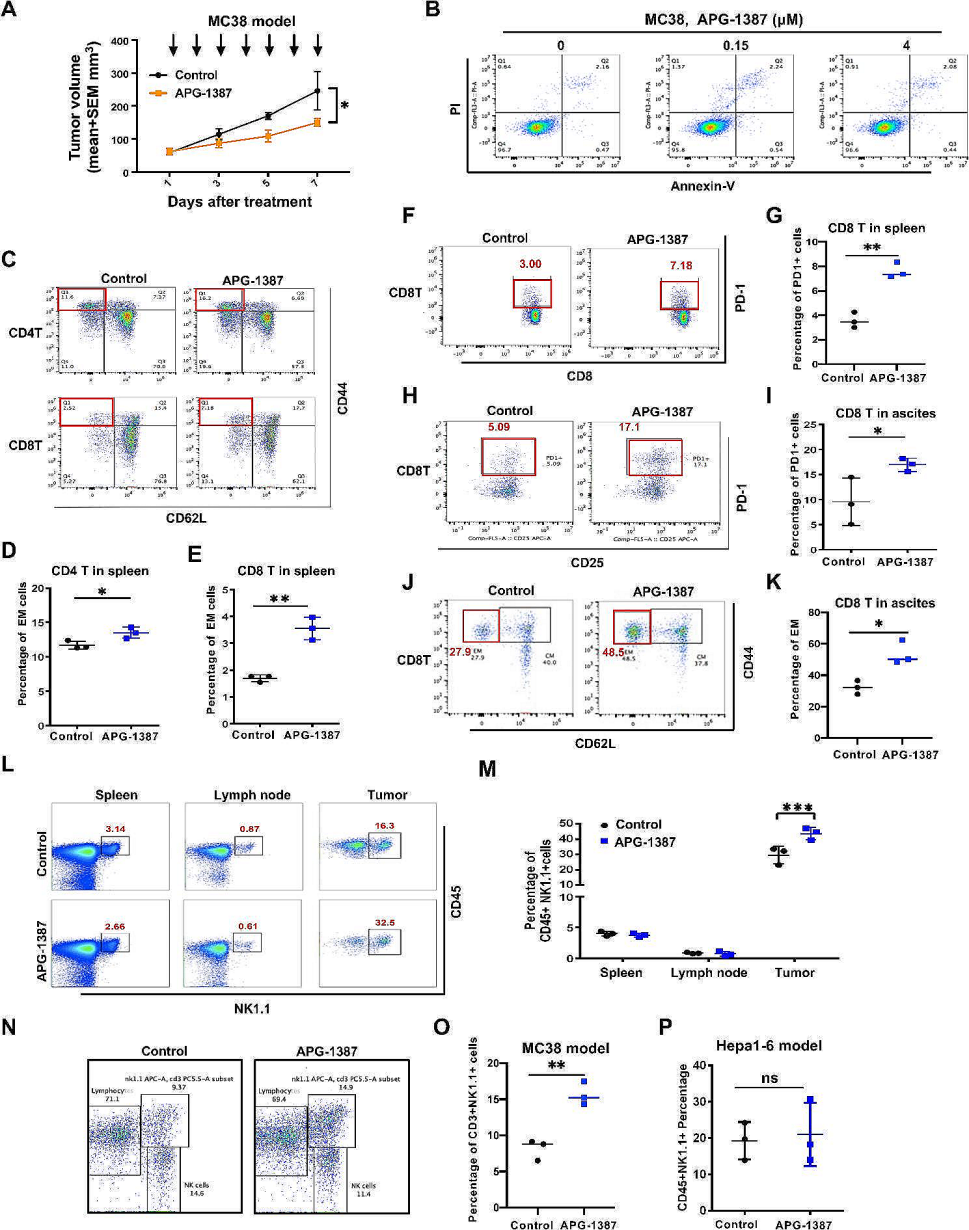
CD3 + NK1.1 + cells in tumor tissue induced by APG-1387 play an important role in the synergistic effect of APG-1387 and anti-PD-1 treatments. **(A)** Tumor volumes of MC38 after treatment with vehicle control and APG-1387 (*n* = 5) were measured at indicated days. Data are shown as mean ± SEM of each group. Statistical analyses were performed using one-way ANOVA, *P < 0.05. **(B)** Annexin V/PI analysis of MC38 cells treated with indicated concentrations of APG-1387 or DMSO for 48 h. **(C)** CD62L-CD44 cells were gated from CD3 + CD4 + cells (*top*)/ CD3 + CD8 + cells (*bottom*) in spleens detected by FACS for vehicle control and APG-1387 groups in MC38 colon cancer models. **D-E.** Statistical analysis of Fig. 3B (left for CD4 + T cells; right for CD8^+^ T cells). **F-G.** PD-1 + cells were gated from CD3^+^CD8^+^cells in spleens detected by FACS for vehicle control and APG-1387 groups in the MC38 colon cancer model. **H-I.** PD-1 + cells were gated from CD3 + CD8 + cells in ascites detected by FACS for vehicle control and APG-1387 groups in the ID8 ovarian cancer model. **J-K.** CD62L-CD44 + cells were gated from CD3 + CD8 + cells in ascites detected by FACS for vehicle control and APG-1387 groups in ID8 ovarian cancer models. **L-M.** CD45 + NK1.1 + cells from spleen, draining lymph node, and tumor harvested from MC38-bearing mice treated with vehicle control or APG-1387 were detected by FACS. A representative image of the two groups is shown. **N-O.** Representative image of CD3 + NK1.1 + cells from MC38 model treated with vehicle control or APG-1387 detected by FACS. **P.** Statistical analysis of CD45 + NK1.1 + cells from tumor harvested from Hepa1-6 (hepatoma)-bearing mice treated with vehicle control or APG-1387 detected by FACS. All the data are presented as the mean ± SD. Statistical analyses were performed using unpaired t test. ns, no statistical difference. *P < 0.05, **P < 0.01, ***P < 0.001

As a single agent, APG-1387 inhibited tumor growth in MC38 models in vivo but had no proapoptotic effect on MC38 cells in vitro (Fig. 3A-B). We thus hypothesized that APG-1387 might play a role in mediating immune-cell functions. To evaluate this hypothesis, we established and implemented additional experiments on APG-1387 treatment in the MC38 model. On the second day after the final APG-1387 treatment, we isolated immune cells from the spleen, draining lymph nodes, and tumors of mice. Nine subsets of immune cells were analyzed between the treatment and control groups, including CD4^+^, CD8^+^, Treg, and NK cells. APG-1387 significantly upregulated the proportions of both effector memory spleen CD8^+^ and CD4^+^ T cells (Fig. 3C-E) and PD-1-positive spleen CD8^+^ T cells (Fig. 3F-G). In ID8 artificial ascites, we also found that APG-1387 upregulated PD-1 expression in CD8^+^ (Fig. 3H-I) and effector memory CD8^+^ T cells (Fig. 3J-K) in the ascites immune cells.

APG-1387 had no effect (vs. vehicle control) on the ratio of immune cell subgroups in the spleen and draining lymph nodes but significantly increased the proportion of CD45 + NK1.1 + cells in tumor tissues (Fig. 3L-M). Further, we figured out that APG-1387 upregulated CD3 + NK1.1 + cells in tumor tissues in MC38 model (Fig. 3N-O). More interestingly, the Hepa1-6 hepatoma model, which had a similar cytokines panel response to APG-1387 as ID8 (but without response to the combination of APG-1387 and anti-PD-1 antibody; Fig. 2F), there was no difference in proportions of CD45 + NK1.1 + cells in tumors between APG-1387 treatment and vehicle control (Fig. 3P). On the basis of these findings, we concluded that APG-1387-induced CD3 + NK1.1 + infiltration in tumor tissues played a critical role in mediating the synergistic effects of APG-1387 and anti-PD-1 antibody treatments.

### IL-12 blockade abrogates the synergistic effect of APG-1387 and anti-PD-1 antibody

**Fig. 4:**
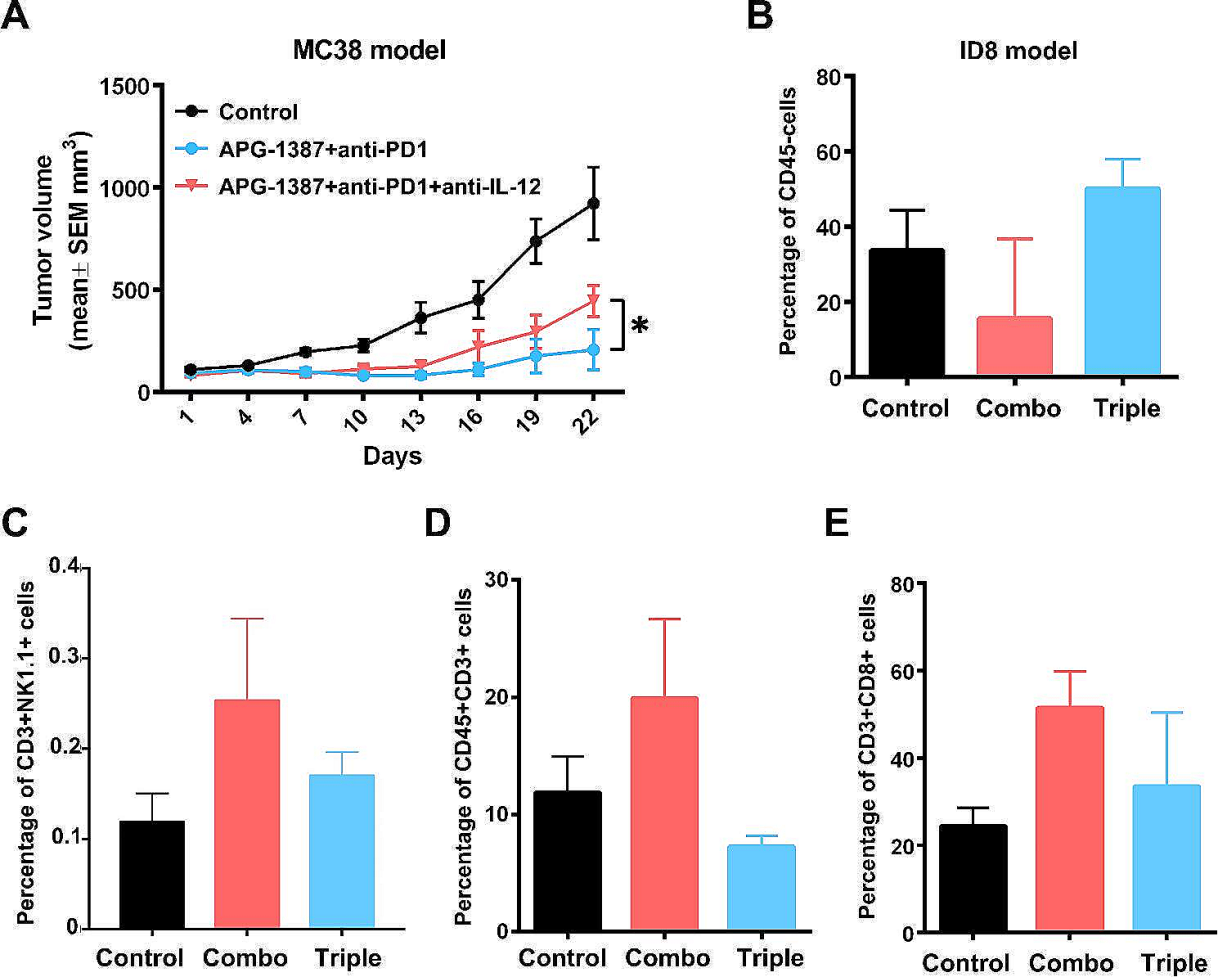
IL-12 blockade reduces the synergistic effect of combined APG-1387 and anti-PD-1 antibody treatment. **(A)** In the MC38 colon cancer model, additional anti-IL-12-p40 antibody (500 µg/mouse) was administrated simultaneously with APG-1387 and anti-PD-1 treatment. Four mice per group. Comparison of vehicle IgG control, two-drugs combination and triple at Day 22 after treatment onset. *P < 0.05 by unpaired t-test. **(B)** CD45- cells were detected in ascites by FACS for three groups in the ID8 ovarian cancer model; triple compared to the two-drugs combination group, *P* = 0.0559 by unpaired t-test. **(C)** CD3 **+** NK1.1 + cells were gated from CD45 + cells in ascites detected by FACS for three groups in the ID8 ovarian cancer model. Triple compared to two-drugs combination group, *P* = 0.204 by unpaired t-test. **(D)** CD3 **+** cells were gated from CD45 + cells in ascites detected by FACS for three groups in the ID8 ovarian cancer model. Triple compared to two-drugs combination group, *P* = 0.0677 for the triple compared to the combination group by unpaired t-test. **(E)** CD8 + cells were gated from CD3 **+** cells in ascites detected by FACS for three groups in ID8 ovarian cancer model. Triple compared to two-drugs combination group, *P* = 0.1716 by unpaired t-test. All the data are presented as the mean ± SD.

What was the relationship between changes in cytokines and tumor infiltration by CD3 + NK1.1 + cells? Among the cytokines, IL-12 was unique because it was produced only by tumors but not T cells. To address whether IL-12 is the key cytokine for CD3 + NK1.1 + cell recruitment, we blocked it by using an anti-IL-12p40 antibody in vivo. With IL-12p40 blockade, we found that the synergistic effect of APG-1387 and anti-PD-1 antibody was reduced in MC38 colon cancer and ID8 ovarian cancer models (Fig. 4A-B). In the ID8 model, adding an anti-IL-12p40 antibody in the triple treatment group tended to downregulate infiltration of CD3 + NK1.1+, CD3, and CD8 T cells compared to the APG-1387 and anti-PD-1 combination group (Fig. 4C-E). Consequently, IL-12 blockade might abolish the synergistic effect of APG-1387 and anti-PD-1 antibody by reducing tumor tissue infiltration by effective immune killing cells.

### The synergistic effect between APG-1387 and anti-PD-1 antibody is mediated by IFN-γ

**Fig. 5:**
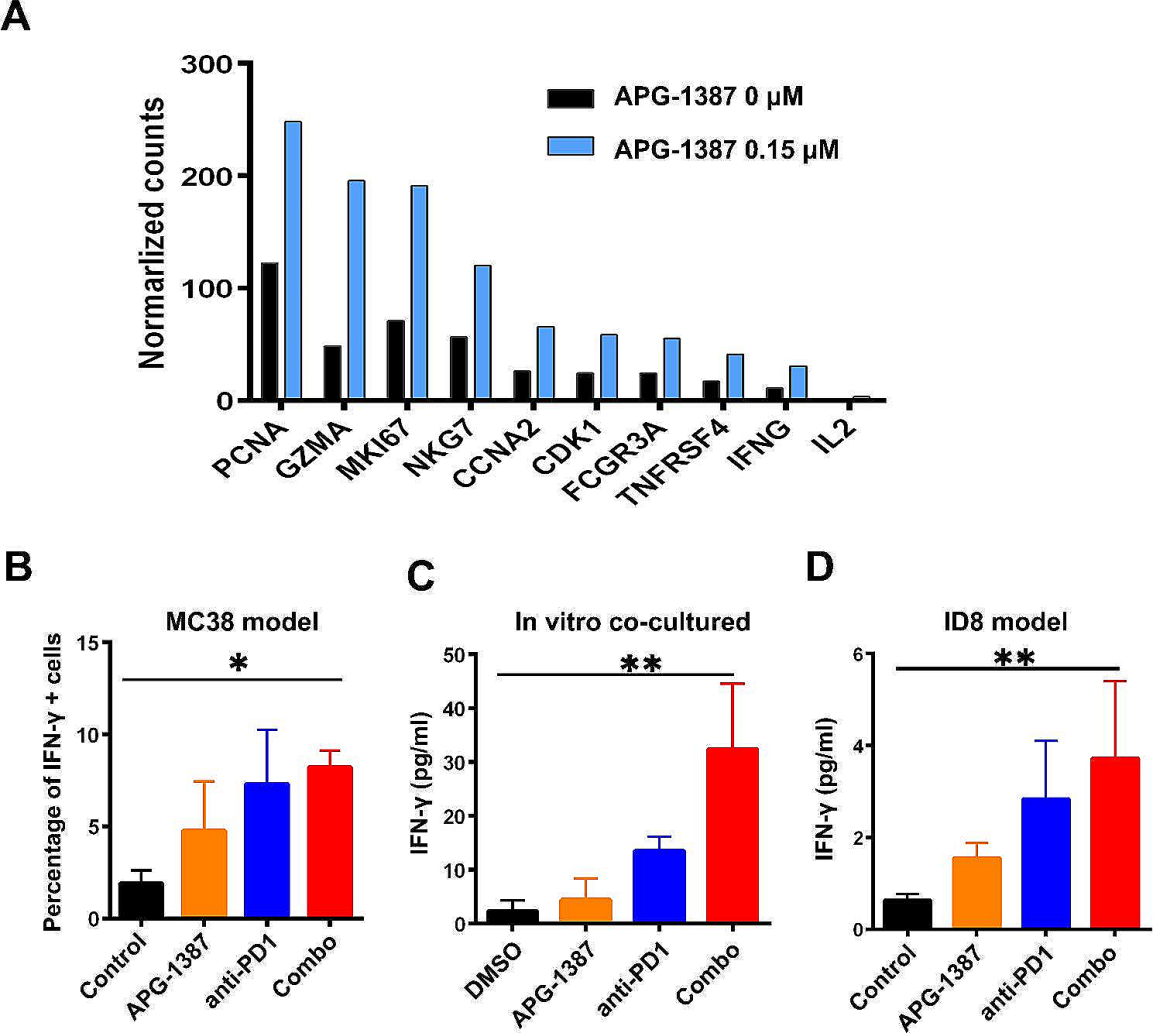
Synergistic effect between APG-1387 and anti-PD-1 antibody is mediated via *IFN-* γ.. **(A)** A total of 10 up-expressing genes were selected from 815 significantly up-expressing genes compared between groups treated with APG-1387 0.15 µM or DMSO control. Sample RNA sequencing from human PBMCs treated with coated CD3 and CD28 stimulation together with 5 ng/mL transforming growth factor-β1 (TGF-β1) and indicated concentrations of APG-1387 for 7 days by the Illumina sequencing platform. DEGseq was used for differential gene expression analysis, and treatment and reference groups were compared. Genes with |log_2_ratio| ≥ 1 and q < 0.05 were selected as significantly differentially expressed genes to obtain the number of up-and-down genes. The GO (gene ontology) functional significance enrichment analysis can determine the main biological functions of differentially expressed genes. After the calculated *P* value was corrected, q < 0.05 was taken as the threshold value and the GO item satisfying this condition defined as a GO item significantly enriched in the differentially expressed gene. **(B)** IFN-γ^+^ cells were detected in tumor-infiltrating lymphocytes by FACS for four groups in the MC38 colon cancer model. **(C)** IFN-γ levels were detected in an *in-vitro* coculture system comprising MC38 and splenocytes from MC38 cured mice (1:20). **(D)** IFN-γ levels were detected in ID8 ascites by MSD for four groups. All the data are presented as the mean ± SD. Statistical analyses were performed using one-way ANOVA. *P < 0.05, **P < 0.01

APG-1387 had a costimulatory effect on activated PBMCs, with RNA expression profiles suggesting a significant upregulation of IFN-γ (Fig. 5A). In the MC38 colon cancer model, APG-1387 stimulated immune cells to secrete more IFN-γ, as demonstrated by flow cytometry (Fig. 5B). Studying *in-vitro* culture system of MC38 and splenocytes from MC38-cured mice with the treatment of IgG, APG-1387, or anti-PD-1 antibody, or the combination of APG-1387 and anti-PD-1 antibody, we found that the combination group produced significantly more IFN-γ (Fig. 5C). Meanwhile, in ID8 artificial ascites, the combination treatment tended to increase IFN-γ levels, according to MSD cytokine detection (Fig. 5D).

## Discussion

Although ICIs have revolutionized the treatment of many malignant tumors, the efficacy is still unsatisfactory in some patients. For example, the MSS subtype, which comprises 95% of patients with CRC, does not respond to these agents.

There are two possible explanations for this phenomenon [5, 22]. First, is endogenous resistance in tumor cells, such as tumor antigen deficiency (low tumor mutation burden). Second is exogenous resistance of these cells, such as T-cell deletions; expression of other inhibitory molecules, such as V-domain Ig suppressor of T cell activation (VISTA), lymphocyte-activation-protein 3 (LAG-3), and T-cell immunoglobulin and mucin-domain containing-3 (TIM-3); and recruitment of tumor-associated macrophages and Tregs.

Surmounting endogenous and exogenous resistance and expanding the population of patients who might benefit from CPIs will likely be the next focus in tumor immunotherapy. Among several efforts to augment PD-1-blocking treatment, changing the tumor microenvironment (TME) from “cold” to “hot” is believed to be the most promising strategy. A previous study showed that Smac mimics promoted multiple myeloma cells with intact noncanonical NF-κB pathway to secrete type I-IFN, thereby inducing phagocytosis of multiple myeloma cells by macrophages [11]. Recently, interim clinical data on IAP inhibitor birinapant combined with pembrolizumab demonstrated that 1 of 12 patients with solid tumors had a confirmed PR to treatment: an individual with MSS CRC. However, as mentioned previously, no efficacy was seen upon treatment with a high dose of birinapant, which was determined by safety consideration [12].

In our study, APG-1387 at low dose conferred immune regulatory effects on certain tumor and immune cells both in vitro and in vivo. Taken together, our findings suggest that a low dose of an IAP inhibitor may sensitize some tumors to anti-PD-1 treatment and save effector cells from death. However, the traditional clinical dose selection dependent on toxicity could lead to selection of high-dose IAP inhibitors when combined with anti-PD-1 CPIs in clinical trials, culminating in trial failure. Therefore, dose selection based on pharmacodynamics, rather than toxicity, is critical for this combination.

In a previous study, we found that APG-1387 up-regulated the expression of HLA-DR in PLC/PRF/5 via activating the non-canonical NF-κB pathway [16]. In our study, we discovered that certain tumor cells could secrete more cytokines essential for T-cell and NK-like cell proliferation and activation during APG-1387 treatment in vitro, and that these tumors were effectively inhibited by APG-1387 and anti-PD-1 antibody in vivo.

Our data indicate that APG-1387 could increase more positive-regulatory cytokines for tumor-killing immune cells. IL-12 is an important growth factor for activated T cells and NK cells that is required for T cell-independent induction of IFN-γ and induces TH1 (helper) cells together with IFN-γ and type I IFN [23]. Simultaneously IL-12 also augment the CD3 + NK1.1 + cells induced from hepatic mononuclear cells [24]. It is mediated by the transcriptional protein signal transducer and activator of transcription 4 (STAT4) activator and is regulated by NF-κB [25]. Recent studies have found that the intestinal flora *Akkermansia muciniphila* restores the efficacy of PD-1 blockade by increasing recruitment of CCR9^+^ CXCR3^+^ CD4^+^ T lymphocytes to mouse tumor beds in an IL-12-dependent manner [26]. It has also been reported that one dose of an IAP inhibitor could stimulate dendrite cells to secrete IL-12 [27]. To our surprise, we have observed that APG-1387 can induce secretion of IL-12 in tumor cells. Conversely, blocking IL-12 in vivo could abrogate the synergistic effect of APG-1387 and anti-PD-1 antibody combination treatment, which suggests that IL-12 plays a key role in this synergetic effect.

Moreover, in our study PD-1 antibody treatment promoted the production of IL-2, IFN-γ and TNFα but did not increase IL-12 level. However, systemic administration of IL-12 is too toxic for human body to terminate those IL-12 related clinical trial. By rising local level of IL-12 in tumor tissue, APG-1387 could resolve the safety concern caused by systemic administration of IL-12. Taken together, we found that IL-12 was a unique cytokine only produced by some tumors, which indicating that the synergistic effect for IAP inhibitor combined with anti-PD-1 is model dependent. IL-12 may be used as a biomarker to predict this synergistic effect.

Our previous study showed that APG-1387 significantly upregulated NK cells in PLC/PRF5 tumors in vivo. Here, in mouse tumor models with responses to combination therapy, more CD3 + NK1.1 + cells were seen in the tumor bed rather than in spleen or draining lymph nodes, indicating that APG-1387 shaped the TME by not only producing multiple cytokines but also further increasing the number of effector tumor-killing immune cells. Obviously, CD3 + NK1.1 + cells mediate the crucial immune modulatory effect of APG-1387 in solid tumors. As we know that CD3 + NK1.1 + cells are commonly called NKT cells and have similar killing function as NK cells, are the first frontier for malignant cell elimination [28], also, importance of the Th1/Th2 differentiation by NKT cells in PD-L1 negative cancer has been revealed recently [29]. Moreover, NKT cells, in addition to T cells, mediate the effect of PD-1/PD-L1 blockade immunotherapy [30, 31].

Consequently, our research indicates that, by increasing more CD3 + NK1.1 + cells in tumors, APG-1387 shows promise in converting “cold” into “hot” tumors and eventually leads to synergistic effects of APG-1387 and anti-PD-1 antibody therapy in certain tumor types and under the appropriate conditions (including an appropriate dose).

## Conclusions

The Smac mimetic APG-1387 modulates multiple aspects of tumor immunity and confers a significant synergistic effect when combined with anti-PD-1 antibody by releasing certain cytokines. APG-1387 upregulated tumor-infiltrating CD3 + NK1.1 + cells by cytokines induced from tumor cells. This study provides solid preclinical evidence for combination therapy of APG-1387 and anti-PD-1 antibodies in clinical trials, especially in tumor type selection and dose selection. These promising but preliminary findings provide a rationale to evaluate the safety and therapeutic effect of this combination in clinical studies, in particular a phase 1/2 trial involving patients with advanced solid tumors or hematologic malignancies (NCT03386526).

### Electronic supplementary material

Below is the link to the electronic supplementary material.

Supplementary Material 1

## Data Availability

The data of this study are available from the corresponding authors on reasonable request. Research Data Deposit number: RDDB2018000471.
