# Supplementary material for: Synergistic effects of Smac mimetic APG-1387 with anti-PD-1 antibody are attributed to increased CD3 + NK1.1 + cell recruitment secondary to induction of cytokines from tumor cells
Source: Cancer Cell Int. 2024 May 24;24:181. doi: 10.1186/s12935-024-03373-7 (PMC11127426; doi:10.1186/s12935-024-03373-7)
Supplement: Supplementary file 1 — Supplementary Material 1 [file 12935_2024_3373_MOESM1_ESM.docx]

Supplementary Materials for

**Synergistic effects of Smac mimetic APG-1387 with anti-PD-1 antibody are attributed to increased CD3+NK1.1+ cell recruitment secondary to induction of cytokines from tumor cells**

Wentao Pan^1, 2*^, Qiuyun Luo^3*^, Eric Liang^2^, Mude Shi^1^, Jian Sun^3^, Huimin Shen^4^, Zhenhai Lu^5^, Lin Zhang^1,6^, Xianglei Yan ^1^, Luping Yuan^1^, Suna Zhou^1^, Hanjie Yi ^1^, Yifan Zhai^2#^, Miao-zhen Qiu^1,7#^, Dajun Yang^1,2#^

^1^Department of Experimental Research, State Key Laboratory of Oncology in South China, Collaborative Innovation Center for Cancer Medicine, Sun Yat-sen University Cancer Center, Guangzhou, China.

^2^Ascentage Pharma (Suzhou) Co, Ltd, Suzhou, Jiangsu Province, China

^3^Department of Clinical Research, The Third Affiliated Hospital of Sun Yat-sen University, Guangzhou, Guangdong, China.

^4^Department of Gynecology, The First Affiliated Hospital of Sun Yat-sen University, Guangzhou, China.

^5^Department of Colorectal Surgery, Sun Yat-sen University Cancer Center, Guangzhou, China.

^6^Department of Clinical Laboratory, Sun Yat-sen University Cancer Center, Guangzhou, China.

^7^Department of Medical Oncology, Sun Yat-Sen University Cancer Center, State Key Laboratory of Oncology in South China, Collaborative Innovation Center for Cancer Medicine, Guangzhou, China.

*These authors contributed equally to this work

^#^Corresponding authors

**Table S1. The detailed information of the antibodies and regents used in this study.**

| Antibodies and regents | Catalog | Company |
| --- | --- | --- |
| Anti-Mouse CD45 alexa four700  Anti-Mouse CD3e PerCP-Cyanine5.5  Anti-Mouse CD4 FITC  Anti-Mouse CD8a PE-Cyanine7  Anti-Mouse CD62L (L-Selectin) PE  Anti-Mouse CD44 APC-eFluor® 780  Anti-Mouse NK1.1 APC  Anti-Mouse CD25 APC  Anti-Mouse IFN gamma APC  Anti-Mouse CD279 (PD-1) PE-eFluor® 610  Anti-Mouse CD274 (PD-L1) PE-Cyanine7  major histocompatibility complex II (MHC-II)  Mouse 1× Lymphocyte Separation Medium  Foxp3 / Transcription Factor Staining Buffer Set  InVivoMab anti PD-1  InVivoMab anti-mouse IL-12 p40  InVivoMab IgG  Annexin V-FITC/PI apoptosis kit | 56-0451-82  45-0031-82  11-0042-82  25-0081-82  12-0621-82  47-0441-82  17-5941-82  17-0251-81  17-7311-82  61-9985-82  25-5982-82  17-5321-82  DKW33-R0100  00-5523-00  BE0146  BE0051  BE0089  100-101 | eBioscience  eBioscience  eBioscience  eBioscience  eBioscience  eBioscience  eBioscience  eBioscience  eBioscience  eBioscience  eBioscience  eBioscience  DAKEWE  eBioscience  BioXcell  BioXcell  BioXcell  GOONIE |
